# Topiramate for Abnormal Eating Behaviour in Frontotemporal Dementia

**DOI:** 10.3233/BEN-120257

**Published:** 2013

**Authors:** Colin Singam, Mark Walterfang, Ramon Mocellin, Andrew Evans, Dennis Velakoulis

**Affiliations:** ^1^Neuropsychiatry Unit, Royal Melbourne Hospital, MelbourneAustralia; ^2^Melbourne Neuropsychiatry Centre, University of Melbourne, MelbourneAustralia

**Keywords:** Frontotemporal dementia, executive dysfunction, hyperphagia, topiramate

## Abstract

Topiramate is a sulfamate-substituted monosaccharide anticonvulsant that is associated with anorexia and weight loss and has been used to treat binge eating disorder and bulimia nervosa. This report describes a man with frontotemporal dementia, behavioural variant, associated with abnormal eating behaviour which appeared to respond to topiramate. We review the physiological basis of abnormal eating behaviour in frontotemporal dementia and explore possible mechanisms of action by which topiramate may modify eating behaviour in this condition.

## 1. Introduction

Topiramate is a sulfamate-substituted monosaccharide anticonvulsant that has been associated with anorexia and weight loss [1] and been found to be of benefit in binge eating disorder [1] and bulimia nervosa [2]. We describe a case of a patient with frontotemporal dementia associated with abnormal eating behaviour which responded to topiramate.

## 2. Case report

A 42 year old man was diagnosed with frontotemporal dementia, behavioural variant (bvFTD) after presenting with a 3-year history of coarsening of personality and disinhibited and poorly judged behaviour, into which he lacked insight. As a result, he had lost his job and social standing. On mental state examination, he presented with psychomotor acceleration, impulsivity and disorganisation. Brain imaging findings demonstrated marked frontal and anterior temporal atrophy, particularly on the right, while neuropsychological testing demonstrated significant executive impairment. The presenting symptoms, signs and investigations met Neary criteria for bvFTD [3].

The patient's family described altered eating behaviour which had led to significant behavioural difficulties. He developed a "sweet tooth," chewing 10 packets of gum per day, eating sweets to excess and drinking litres of soft drink. In spite of food being hidden, he continued to pursue sweet foods. He asked strangers for money so he could buy sweets and he would take food that was not his at social gatherings. He tended to eat at a rapid pace and eat too much, often to the point of vomiting. This was the case with all foods, not just sweets. His wife described a significant weight increase of more than 10 kg.

The patient was commenced on quetiapine soon after admission with a resultant improvement in his psychomotor agitation and challenging behaviours but without any alteration in abnormal eating behaviour. His weight increased from 78.2 kg to 81.5 kg over four weeks. He stole food from other hospital patients'

ISSN 0953-4180/13/$27.50 © 2013 – IOS Press and the authors. All rights reserved plates, especially desserts. His wife complained that his food seeking was the most difficult behavioural challenge remaining following a trial of weekend leave.

Topiramate was commenced at 25 mg bd and the dose titrated to 200 mg bd but reduced to 100 mg bd due to nausea. An improvement was noted in his eating behaviours within 3 weeks of the commencement of topiramate. He ate more slowly and did not overeat to the same extent as previously. He continued to overindulge in sweet foods when they were available but would not seek out sweets with the same persistence. His wife continued to hide sweet foods in the house but other foods were returned to open access. His weight noticeably reduced, from 81.5 kg to 72 kg over six months following the commencement of topiramate.

## 3. Discussion

We demonstrated that topiramate improved abnormal eating behaviour in bvFTD when other pharmacotherapy directed at challenging behaviours had failed. Abnormal eating behaviour in bvFTD represents a significant management challenge. The mechanism of abnormal eating behaviour in bvFTD is not fully understood but it has been proposed that dementia-induced changes in the right orbitofrontal and ventral insular cortex and striatum play a role [4]. This network is involved in regulating complex behaviour and alterations in this network have been described in bulimia nervosa [5]. Topiramate has been reported to ameliorate bingeing in bulimia nervosa [2] although its exact mechanism of action is unclear. There is also evidence of reduced volume in the posterior hypothalamus in bvFTD, particularly in patients with significant abnormal eating behaviour [6].

Topiramate is an antagonist of glutamatergic receptors alpha amino-3-hydroxy-5-methylisoxozole-4-propionicacid (AMPA) and kainate. Antagonism of AMPA has been associated with reduced reward seeking behaviour in animal models of substance dependence [7]. Whilst post-mortem studies show that AMPA activity is reduced in the frontal and temporal lobes in bvFTD [8], it may be that topiramate's antagonism of glutamatergic activity in the lateral hypothalamus activates its satiety centre and thus reduces appetite [1]. Topiramate also augments GABAergic transmission, although this is unlikely to reduce appetite, as increased hypothalamic GABAergic transmission is not associated with attenuated eating behaviour [9].

Other explanations may account for the observed improvement. Topiramate's effect may have been related to a general action on the patient's compulsive behaviours rather than a specific effect on drive for eating, although these behaviours had already resolved significantly when quetiapine was instituted. Environmental factors, such as altering access to food, might contribute to the apparent change in eating behaviour. Finally, the behavioural disturbances of bvFTD can spontaneously resolve over time. Nonetheless, successful weight reduction in this case report suggests that topiramate should be considered in patients with abnormal and challenging eating behaviours related to bvFTD. Our findings warrant replication in group studies of the disorder, which may clarify whether topiramate's effects are restricted to eating behaviours or if this medication has broader utility in managing behavioural dyscontrol in bvFTD.
